# Deaths and clashes induced by rivalry among fans dur-ing FIFA World Cup 2022 in Bangladesh

**Published:** 2024-07

**Authors:** S. M. Yasir Arafat, Reinhard Heun, Mohammad Sorowar Hossain

**Affiliations:** ^*a*^ Biomedical Research Foundation, Dhaka, Bangladesh.; ^*b*^ Medical School, University of Bonn, Germany.

**Keywords:** Football World Cup, Soccer, Sports, Fans, Social emotion, Rivalry

## Abstract

**Background:**

International sports events like the Football World Cup affect the collective emotions of the global community. Usually, major sports events like the Football World Cup show beneficial effects on the community. However, many unwanted consequences such as accidents, premature deaths and violent supporter rivalries have been noticed during it. We aimed toreport preventable premature deaths and clashes induced by rivalry among fans during the FIFA World Cup 2022 in Bangladesh.

**Methods:**

We monitored local newspaper reports for data collection during the World Cup 2022 event and collected data for age, sex, the possible cause of death, and precipitating events related to the event.

**Results:**

We found 23 World Cup Football-related deaths, 35 hospitalizations, and 45 injuries related to clashes between the rival fans of Argentina and Brazil. The median age of deceased was 20 years. The majority of the deaths happened in young males due to fall while hoisting flags. Deaths in the late age happened due to sudden emotional upsurge during the game day. And, clashes between rival fans mostly happened due to social bullying favoring and disfavoring a team.

**Conclusions:**

This report indicates an important area of potentially preventable death related to World Cup Football and warrants public health attention even in a non-participating country, both locally and internationally.

## Introduction

Football is the most popular game affecting billions of people worldwide. One recent systematic review identified that although some components like the sense of inclusion, shared purpose, and positive peer identification act as components of resilience, ultimately it does not affect the mental health of spectators.^1^ On the other way round, unjustified passion, emotion, and loyalty with fanaticism in supporting a team can produce harmful effects on physical health, and mental well-being and even end the life.^1^

International sports events affect the collective emotions of the global community. Emotional arousal, particularly during high-stakes international sports events, plays a significant role in shaping fan behavior. The intensity of emotions such as joy, frustration, and anger can lead to extreme actions, including violence or self-harm.^2^ Group identity also amplifies this effect, as fans often associate themselves with their chosen team, treating its successes and failures as personal.^3^ It has been noted that public ceremonial events increase social integration, reduce suicidality.^4^ Previous reports identified that winning a team in the World Cup football reduced suicide in France in 1998 and exiting a team from the group stage increased suicidal behavior by overdose in Iran in 2014.^5,6^ A reduction in self-harm was also noted among males in Scotland during the Football World Cup tournaments possibly due to distraction from personal problems with more enthusiasm for the country.^7^ Again, an increased rate of suicidal behavior was noted after a defeat at the home of the local football team in Ukraine.^8^ These incidences were noted among the residents of the country participating in major sports events. However, we also noted suicides among football fans of the Argentinian teams in residents of non-participating countries (Bangladesh and India) when their team lost.^9^

Bangladesh, a South Asian country with a population of nearly 170 million, has never qualified for the final stage of the World Cup football event and it has no chance in the near future as it is in the tail end of the world’s football ranking (192). Yet, surprisingly, people of this country are enthusiastically supporting two Latin American football-playing countries- Argentina and Brazil. While intense emotional responses among fans are common worldwide, the specific cultural and social context in Bangladesh, where football is followed with fervor despite the country not participating in the World Cup, may amplify these reactions. The deep-rooted passion for foreign football teams like Argentina and Brazil, fostered by decades of media exposure and cultural icons, heightens the emotional stakes for Bangladeshi fans.^9^

Football’s popularity in Bangladesh increased since the mid-1980s when televisions became widely available in the country. Argentina became a very popular team in 1986 when Diego Maradona won the FIFA World Cup title. The enthusiasm for the Argentinian team has risen to a new level because of Lionel Messi. On the other hand, Pele played a key role in developing fans for Brazil as his biography was included in textbooks at the primary school level in the 1970s and 1980s. The Fédération Internationale de Football Association (FIFA) has noted that Bangladesh has ‘some of football's most passionate fans’.^10^ Even we noted clashes between the Bangladeshi fans of Argentina and Brazil in a refugee camp in Italy.^11^

Hence, every four years, World Cup fever sweeps across Bangladesh.^12^ Fans display their football zeal by hoisting flags on rooftops, painting graffiti of favorite players on walls, and also painting whole buildings in Brazilian or Argentinian national flag colors, showcasing very long flags (these could be over one kilometer in length), mass buying of team jerseys.^13^ During the world Cup, football dominates public discourses and the rivalry sparks a flurry of Brazil vs Argentina football matches in every community, educational institute, offices, and even among family members. In Bangladesh, where football fandom is deeply embedded in cultural identity, these factors can drive extreme behavior. Furthermore, social media has escalated these emotions by providing an immediate platform for fans to express their views and participate in rivalries, often leading to cyberbullying and, at times, real-world confrontations.^14^

In recent years, the popularity of social media (Facebook in Bangladesh) has made engagement among fans easier. Consequently, the risk of cyberbullying among emotional fans has also increased substantially, which, may end up in physical clashes in real life. Against this backdrop, we aimed to report and discuss preventable premature deaths and clashes induced by rivalry among fans during the FIFA World Cup 2002 in Bangladesh.

## Methods 

An article reported suicide among fans during the Football World Cup 2018.^9^ For the current study, we monitored local newspaper reports for data collection during the World Cup 2022 event. The looked for deaths and injuries physical clash and attack and/or any physical events like chest pain or heart attack. The newspaper reports were cross-checked and verified when multiple reports of the same event were available. Data for age, sex, the possible cause of death, and precipitating events for each case were extracted from the news reports. We manually calculated the number of deaths, hospitalizations, and injured persons resulted from accidents and physical clashes. We considered the cases as related to the Football World Cup when the news reports clearly indicate. Usually, the reports mentioned the details of the deaths. We relied for the measures for hospitalization criteria and other-injuries based on newspaper reports. We categorized an incident as 'football-related' if it was explicitly mentioned in the news report that the event was triggered by or occurred in the context of a football match or related activities, such as flag hoisting, celebrations, or altercations between rival fans. The reports were cross-referenced with other available sources whenever possible to ensure accuracy. For example, when multiple news outlets reported the same incident, we compared the information for consistency regarding location, cause, and individuals involved. Data were prepared and presented using Microsoft Excel version 10 for Windows. Descriptive analysis of data was performed. We utilized publicly available news reports which did not involve direct interactions with human subjects. The information gathered was already in the public domain, and no identifying personal details were collected beyond what was reported by the media. This type of analysis does not typically require ethical review since it does not involve intervention or manipulation of subjects. Therefore, we did not seek formal ethical approval. 

## Results

We found incidents of 23 World Cup Football-related deaths, 35 hospitalizations from 45 injured patients among Football fans (Table 1 ). There were ten injured persons without hospitalization. The median age was 20 years ranging from 10 to 55 years for deaths. The deaths happened between November 06 and December 20, 2022, in different districts of the country mentioned as Bagherhat, Barisal, Bhola, Chandpur, Chittagong, Cox's Bazar, Cumilla (2), Dhaka (3), Faridpur, Gaforgaon, Habiganj, Jamalpur, Jessore, Khagrachori, Kustia, Lakshmipur, Naogaon, Netrokona, Sylhet, and Tangail. We found the precipitating factors in the four categories mentioned as hoisting flags (30.4%), heart attack (26.1%), murder (21.5%), and others like accidents (21.5%) for deaths. Deaths related to flag hoisting (n=7) happened to falling from heights while hoisting the flags of supporting countries, cardiac events happened in emotional events of the game (n=6), and murders (n=5) happened due to altercations between supporters of Argentina and Brazil mostly, and accidents during celebration and watching matches. Physical injuries and hospitalizations happened due to altercations and quarrel among the supporters. 

**Table 1:** Profile of reported death cases and clashes during the FIFA World Cup 2022 in Bangladesh.

Variables	N	%	How the incidence triggered
Gender for reported death cases			
Male	23	100	
Female	0	0	
Median age (IQR) in years	20 (22)		
Reported cause of deaths			
Hoisting flags	7	30.4	Over enthusiasm to show support for favorite teams (Mostly Brazil and Argentina)
Heart attack	6	26.1	Excessive mental stress (losing match or during a critical phase of games)
Murder	5	21.7	Bullying and altercation among rival fans while watching matches and stabbed to deaths
Others (accident)	5	21.7	Accident during watching and celebration
Other related issues			
Number of injuries due to clashes among rival fans			
Total Injured cases	45	Bullying, altercations and clashes among emotional rival fans	
No of hospitalized cases	35		

## Discussion

**Premature loss of life**

This study revealed 23 World Cup 2022-related deaths, 35 hospitalizations, and 45 injured persons among the fans of Argentina and Brazil. No death among females was noted. The majority of the deaths happened due to flag hoisting which happened in young males. Deaths in the late age happened due to sudden emotional upsurge mentioned as cardiac events. And, clashes between rival fans mostly happened due to bullying related the supported team. The findings indicate the level emotional engagement of fans in a non-participatory country in the Football World Cup. In this case, the emotion has been expressed in a negative way, injury and loss of life. The other notable finding is that all the reported cases happened in males which can be explained by enduring gender role in Bangladesh. One previous study found that greater than a two-fold increase in acute cardiovascular events among supporters who watched a stressful football World Cup match. The risk was excessive among males with known coronary artery disease.^15^ Preventive measures focusing on the risky populations were recommended. 

**World Cup-related deaths and clashes: Role of Sociology of Emotion**

Deaths and clashes that took place during FIFA World Cup 2022 in Bangladesh are deeply embedded in the sociology of emotion. Emotions are more evident for those social events including sports are changed with lasting emotions and passions.^16^ Many events of sports essentially carry the enormous weight of the emotional load and develop a stringent emotional bondage among the fans.^17^ Violence, intimidation, and excitement are the critical reflections of heightened emotions and the deepest engagement of the fans, often stimulated by several social circumstances.

**Implications of the Study Results**

The study indicates potentially preventable deaths related to World Cup Football (2022) and warrants public health attention even in a non-participating country, both locally and internationally. Awareness among parents and social leaders about flag hoisting at risky places can prevent the youths fall. Additionally, for the elderly persons with heart diseases could avoid their emotional engagement to prevent cardiac events. Awareness in the community can prevent Preventive messages for unwanted deaths should be broad-casted while promoting the WC events on behalf of FIFA as well as the individual country. Family members perhaps could play the most important role as they already know the potentially vulnerable persons and possible ways of restriction the person. 

To mitigate the risks associated with extreme fan behavior, specific preventive measures could be implemented at both community and national levels. Educational campaigns targeting young people could raise awareness about the dangers of overzealous fandom, especially regarding risky activities like flag hoisting in unsafe areas. Community outreach programs led by local leaders, sports clubs, or schools could foster healthier expressions of fandom by promoting sportsmanship and non-violent rivalry. These initiatives could include discussions on managing emotions during high-stakes matches and the responsible use of social media to prevent cyberbullying and its escalation into physical confrontations. Additionally, local authorities could implement restrictions on flag hoisting in high-risk areas, such as rooftops, to prevent fatal accidents. Public health campaigns could also focus on encouraging fans with known cardiovascular risks to avoid high emotional stress situations during critical match moments.

**Strengths and Limitations**

This is the second analysis after the Football World Cup 2018 assessing Football World Cup -related unnatural deaths. We assessed media reports published in multiple mainstream media with a nearly similar story. While this study sheds light on the extreme behaviors of football fans during the 2022 World Cup, several limitations should be considered when interpreting the findings. The reliance on media reports may have led to underreporting of incidents, especially in rural areas where media coverage is less comprehensive. Additionally, media reports may not always capture the full context or intricacies of each event, such as underlying psychological or social triggers, which limits our ability to fully understand the causes of these incidents. It is also challenging to isolate football-related incidents from other ongoing societal factors that may have contributed to clashes or emotional distress. These limitations reduce the generalizability of the findings and suggest the need for more comprehensive data collection methods in future research, such as direct surveys or interviews with affected individuals. We did not assess the psychological and/or social factors of the deceased or victims. 

## Conclusion

This study revealed the adverse outcomes of extreme emotion among football fans in a non-participating country during the Football World Cup 2022. It warrants public health attention from local and international agencies to prevent the events. Family members and the surrounding society could play vital roles in preventing such events. 

**Acknowledgment**

We thank Anisur Rahman Khan for his comment on the manuscript.

**Author Contribution**

Conceptualization: Mohammad Sorowar Hossain

Methodology: S. M. Yasir Arafat and Mohammad Sorowar Hossain: Project Administration: Mohammad Sorowar Hossain: Supervision: SM Yasir Arafat

Writing – Original Draft Preparation: All authors

Writing – Review & Editing: All authors

All authors have read and approved the final version of the manuscript.
